# High prevalence of toxigenic *Clostridium difficile* in public space lawns in Western Australia

**DOI:** 10.1038/srep41196

**Published:** 2017-02-01

**Authors:** Peter Moono, Su Chen Lim, Thomas V. Riley

**Affiliations:** 1 Microbiology & Immunology, School of Pathology and Laboratory Medicine, The University of Western Australia, Nedlands, WA, 6009 Australia; 2 School of Medical & Health Sciences, Edith Cowan University, Joondalup, WA, 6027 Australia; 3 School of Veterinary & Life Sciences, Murdoch University, Murdoch, WA, 6150 Australia; 4 PathWest Laboratory Medicine (WA), Nedlands, WA, 6009 Australia

## Abstract

*Clostridium difficile* is a well-established hospital pathogen. Recently, it has been detected increasingly in patients without hospital contact. Given this rise in community associated infections with *C. difficile*, we hypothesized that the environment could play an important role in transmission of spores outside the hospital. Lawn samples (311) collected in public spaces in the metropolitan area of Perth, Western Australia, from February to June 2016 were cultured for *C. difficile. C. difficile* was isolated from the samples by direct and enrichment culture, and characterized by standard molecular methods using toxin gene PCR and ribotyping. The overall prevalence of *C. difficile* was 59%, new lawn (≤4 months old) was twice as likely as old lawn (>4 months old) to test positive (OR = 2.3; 95%CI 1.16–4.57, *p* = 0.015) and 35 *C. difficile* ribotypes were identified with toxigenic ribotype 014/020 (39%) predominating. The highest viable count from lawn soil samples was 1200 CFU/g. These results show that lawns in Perth, Western Australia, harbor toxigenic *C. difficile*, an important finding. The source of lawn contamination is likely related to modern practice of producing “roll-out” lawn. Further work should focus on identifying specific management practices that lead to *C. difficile* contamination of lawn to inform prevention and control measures.

*Clostridium difficile* is a well-established hospital pathogen associated with outbreaks of severe gastroenteritis^1^. *C. difficile* infection (CDI) in patients is acquired by oro-fecal ingestion of spores from the environment. The major virulence factors of *C. difficile* are two exotoxins, toxins A and B^2^, with some strains producing a third binary toxin^3^. There has been an increase in the incidence of community-associated CDI (CA-CDI) both in Australia^4^ and elsewhere^5^. Because of this increase in CA-CDI^4^,^5^,^6^, food sources have emerged as a potential reservoir of *C. difficile*. This paradigm is supported by studies that have reported detection of *C. difficile* in animals^7^, meat^8^,^9^, and root^10^, and leaf^11^ vegetables. However, the overall prevalence of *C. difficile* in foods is low^12^. Although foodborne transmission of CDI is plausible it has never been proven, and the environment may be another important source of CA-CDI^13^,^14^. This is evident from many studies where various ribotypes (RTs) of *C. difficile* have been detected from the environment including so-called hypervirulent strains (RT 001, 014, 027, 045, 066, 078, and 126)^15^,^16^,^17^. It is possible that environmental contamination with *C. difficile* spores may be a more important source for CA-CDI than food^12^.

Outbreaks of infectious organisms, other than *C. difficile*, associated with manure have been reported^18^,^19^,^20^,^21^. In the USA, 50% of all biosolids produced each year is used in agriculture and landscaping^22^,^23^,^24^. *C. difficile* has been isolated from raw and treated human biosolids^16^, and the environment^15^. The mesophilic treatment of biosolids does not reduce *C. difficile* spore load^25^,^26^ and, therefore, application of biosolids to landscaping including private and public space lawns could transmit *C. difficile* in the community. In Perth, Western Australia, the construction of new housing and the development of new suburbs has seen an expansion of public and private lawns as has occurred in the USA^27^.

The aim of this study was to determine the prevalence and concentration of *C. difficile* in newly established (NL) and older (OL) lawns in public spaces in Perth, and to characterize any *C. difficile* isolates by phenotypic and genotypic techniques. Second, we investigated factors such as the location and size of the lawn to see if they could predict *C. difficile* status.

## Results

### Prevalence of *C. difficile*

The overall prevalence of *C. difficile* in lawn samples was 59% (182/311) varying from 0% to 70% by postcode. The prevalence of *C. difficile* was significantly higher in NLs 65% (129/198) compared to 47% (53/113) in OLs (Table 1). In the unadjusted univariable model, *C. difficile* was more likely to be detected in NL than OL (OR = 2.11, 95% CI: 1.32–3.4, *p = *0.001) compared to the adjusted model (OR = 2.30, 95% (CI: 1.16–4.57, *p = *0.015) (Table 1). In the multivariable analysis, age of lawn was also the only variable that was significantly associated with detection of *C. difficile* (OR = 1.92, 95% (CI: 1.15–3.21, *p* = 0.013). The size and location of the lawns were not associated with detection of *C. difficile* and the study period was too short to observe any effect of season on recovery.

### Quantitation of *C. difficile* in lawn

Viable counts were performed on 10 lawn samples taken from one positive area by direct culture on C.diff ChromID agar (Table 2). Samples were also concentrated by centrifugation. Using PBST or PST diluent, the highest number of *C. difficile* positive cultures was 8 out of 10 after centrifugation. *C. difficile* recovery was higher in PST than PBST by both direct culture (50% vs 40%) and after centrifugation (80% vs 60%). The highest viable count of *C. difficile* detected was 1200 CFU/g of soil and the lowest 50 CFU/g of soil (Table 2). There were three ribotypes detected, all non-toxigenic, QX 189, QX 601, and UK 010.

### Molecular characterization

There were 35 unique RTs detected in this study. Of the toxigenic (A^+^ B^+^ CDT^−^) RTs detected, 11 isolates harboured *tcdA* and *tcdB* genes but no binary toxin genes (Table 3). Toxigenic RTs accounted for 47% (86/182) of the total and included internationally recognized RTs associated with hospital infections. The 24 non-toxigenic (A^−^ B^−^ CDT^−^) RTs accounted for 53% (96/182). The toxigenic strain RT 014/020 was the most predominant (39.0%), followed by the non-toxigenic RT 010 (20.3%) (Table 3). Toxigenic RTs isolated from NL accounted for 33.5% (61/182) of all isolates compared to 14.3% (26/182) in OL. In the NL among toxigenic strains only, RT 014/020 was over represented 78.7% (48/61), followed by RTs 054, 056, 002, 018, QX 610, and QX 611 (all <5%). In the OL among toxigenic strains only, RT 014/020 was also over represented at 88.5% (23/26), followed by RTs 002, 106 and QX 409 (all <5%).

## Discussion

The aim of this study was to investigate the prevalence of *C. difficile* in public space lawns in Perth, Western Australia. The reasons for undertaking the project were three-fold. First, there has been an increase in the incidence of CA-CDI in Australia^4^. Second, although many production animals carry *C. difficile* in their gut, particularly young animals, it is unlikely that meat contamination plays a major role in CA-CDI^12^. Last, anecdotally we learned that manure from pig farms was being used by turf farms for the production of lawn.

The overall prevalence of *C. difficile* in lawn samples was 59%, varying from 0% to 70% by postcode. In the adjusted univariable model, *C. difficile* was more likely to be detected in NL than OL (OR = 2.30, 95% CI: 1.16–4.57, *p* = 0.015) (Table 1). Other studies have reported a high prevalence (30–68%) of *C. difficile* in the household environment and biosolid treatment plants^15^,^16^, similar to this study. The reason for the lower prevalence of *C. difficile* in OL (>4 months) is not clear, although an effect of natural exposure of germinated spores to oxygen or ultraviolet light cannot be excluded. It is unlikely that *C. difficile* can multiply to any great extent in the lawn and the counts detected were not very high. A recent study has shown that ultraviolet light devices can be used as sanitizers in hospitals to reduce pathogenic organisms including *C. difficile*^28^. Another study found that *C. difficile* spores could survive up to ~5 months in the environment^29^. Therefore, the difference in prevalence between NL and OL could be explained by environmental factors such rain, wind, and ultraviolet light over time, and contact with people and animals which could help disperse the initial spore load, particularly on the surface of the lawn. The majority of the NL in this study was located within recently built suburbs in Perth. The relationship between new suburb buildings and lawn expansion is well studied^27^. Our findings suggest that new suburbs and consequent expansion of NL could be contributing to dispersal of *C. difficile* in the environment and variables other than NL were not significantly associated with isolation of *C. difficile*.

Nearly 25% of global disease burden has been attributed to environmental risk factors^30^. Animal manure is widely used in both agriculture and landscaping^22^,^23^,^24^,^31^, and anecdotal evidence suggests this practice is widespread in Australia. In the USA, manure that has been composted is exempted from federal or state rule regarding pathogen levels if it is going for land application^31^. The major problem which is often overlooked in applying animal manure or biosolids to land is consideration of the duration of persistence of pathogens on plants^31^. Animal effluent treatment involves removal of solid wastes and then holding liquid effluent in anaerobic ponds under mesophilic anaerobiosis. The treatment method for human raw effluent in Switzerland involved grid separation, primary sedimentation, and secondary biological treatment (an activated sludge process)^16^. The major problem in animal manure treatment is lack of separation of feces from young animals versus older animals. Young animals are associated with shedding high levels of pathogenic organisms including, and in particular, *C. difficile*^8^,^31^,^32^. Currently, there is pressure on the turf industry to meet the demand for lawns in newly built suburbs both private and public lawns. This has led the industry to develop the lawn farming system of roll-out lawns which can easily be transported to clients on demand. The lawn, like other plants, is grown on manure or biosolids for rapid growth and early maturation. The fact that even composted manure or treated animal effluent does not inactivate *C. difficile* spores implies that lawns could be heavily contaminated with *C. difficile* and our data suggest this is the case. Even compost, manure or biosolids that have been produced with specific standards^23^ have caused disease outbreaks in the USA^18^,^19^,^20^,^21^. In this regard, manure, compost and biosolids should be screened for pathogenic microorganisms such as *C. difficile* before being applied to land for recreational or agricultural use.

Although the infectious dose for *C. difficile* is unknown, there are suggestions that it could be low (100–1000 spores)^33^. In addition, in some high-risk individuals infection could be driven mainly by host factors. In this study, we found that the highest *C. difficile* spore concentration in the lawn samples was 1200 CFU/g of soil (Table 2). The concentration of *C. difficile* in feces of cattle has been reported as 2.5 × 10^4^ CFU/mL^9^ and in human feces as 6.66 + 0.62 log10 CFU/mL^34^. It is unclear how much exposure to lawns is required for a human or animal to be contaminated with sufficient spores that could lead to infection with *C. difficile.* The fact that ingestion of *C. difficile* from the environment does not quickly result in infection is due to the complex nature of the pathogenesis of disease that requires the gut microflora to be perturbed, usually in the form of antibiotic exposure^5^. Although we did not find any seasonal fluctuations of *C. difficile* in lawn samples in this study, it is likely that recreational exposure to *C. difficile* could be higher in some seasons when people regularly use public spaces and lower in low activity periods. Therefore, studies that quantitate spore load on fomites such as shoes are needed because they may help explain the rise of CA-CDI.

There were 35 unique RTs detected in this study, including the clinically important RTs 014/020, 056, 054, 002, 018, 012 and 043 (Table 3). The high prevalence of RT 014/020 in this study was an interesting and important finding given that RT 014/020 is the most predominant RT in laboratory samples from diarrheic patients in Australia^6^,^35^,^36^. RT 014/020 is also the main ribotype currently found in pigs in Australia^37^ suggesting that pig manure may be responsible for the contamination of lawn in Perth. Other researchers have reported detection of RT 014/020 in river water^17^ and treated sludge^16^.

The detection of RTs 012, 002, and 018 was unexpected because they are not routinely detected from the environment^16^,^17^, although they are increasingly associated with CA-CDI^35^,^36^. Recently, RTs 056, 010, 078, 213, 009 and 020 were detected in dogs^38^. RT 056 was also recently detected in gastrointestinal tracts of veal calves at slaughter^32^. Several of the other RTs detected in our study (056, 054, 018 and 002) have been associated with human CDI in Europe^39^, Japan^40^ and Australia^6^. These findings suggest that all lawns may be getting contaminated with animal strains of *C. difficile* and that this could be contributing to an increase in CA-CDI.

Interestingly, no binary toxin producing *C. difficile* isolates were cultured in this study although such strains have been recovered previously in high numbers from production animals in Australia^32^,^37^,^41^. The reasons for this are unclear. Our earlier animal studies have suggested that different strains of *C. difficile* tend to infect/colonise herds of animals in particular geographic areas^32^,^37^ and these are not always binary toxin producers, such as RT014/020 in pigs^37^. The lack of binary toxin producers in the current study may just reflect a relatively small sample size and the fact this study was undertaken in Western Australia only.

The lawns sampled were located within the metropolitan area of Perth, suggesting that people and dogs walking on lawns could transfer *C. difficile* into the household^42^. A high prevalence (32.3%) of *C. difficile* in household environs has been reported, particularly on the soles of shoes^15^. It is also possible that shoes could be vehicles by which *C. difficile* could be introduced in health care facilities. The detection of *C. difficile* in lawn could help explain the substantial proportion of cases (45%) of CDI originating from unknown reservoirs as recently described by others^43^,^44^.

This study has some limitations. First, we only sampled a relatively small number of public spaces (n = 20) and the study was undertaken only in Western Australia which may impact its generalisability to other regions of Australia and the world. However, all lawn/turf producing companies have access to the same sources of manure in Western Australia, suggesting that the problem could be similar throughout the metropolitan area of Perth. The age of some of the lawns was estimated with a series of photos of lawns with a known age and this could have resulted in misclassification bias. However, this would not have affected the overall outcomes of the study as both OL and NL had relatively high prevalence of *C. difficile*. We investigated management practices of commercial lawn farms, however, the frequency of maintenance of the lawns was not established.

In conclusion, the high prevalence of *C. difficile* in lawn is potentially an important finding, however, it is difficult to estimate the risk of CDI through exposure to *C. difficile* in lawns. There is a need for further detailed and structured studies to examine how modern lawns are grown, and to investigate the role of any manure and biosolids applied as fertiliser as a source of CA-CDI. The relevance of these findings should be further confirmed by studies comparing the relatedness of isolates in this study to those CA-CDI cases using whole genome sequencing.

## Methods

### Background

Lawn in Western Australia can either be raised as seeding lawn or “instant turf” in the form of rolls of pre-grown lawn^45^. Seeded lawn has lower cost to plant compared to instant turf, however, it takes longer to mature and is more labour intensive than turf rolls. The advantage of turf rolls is that they are fast to grow and easy to lay, and can be used within a week, hence the current high demand. Soft leaved buffalo grass is a widely used variety in Western Australia because it can withstand drought; most lawns are established in early autumn or late summer to avoid extreme temperatures in summer^45^.

Lawns in Australia are considered young up to 4 months by the turf industry^45^. Therefore, we used this time-point to distinguish between newly established (NL) and older (OL) lawns [NL (≤4 months) and OL (>4 months)]. From autumn through winter, older lawns tend to lose colour and become dormant. We initiated this study during this period when it should have been relatively easy to identify lawns that were new.

### Study location and samples

NL and OL samples were collected from 20 public spaces within 11 postcodes in Perth and surrounding areas in February–June 2016. When information on the age of lawns in some areas was unavailable, photographic identification was employed. A NL was identified by the presence of a visible line between two or more strips of laid down lawn which remained visible for up to 4 months. Lawns that did not have these were assumed to be old^45^. This involved comparing photographs with a series of photographs taken of lawns of known age (NL appeared bright green, luxuriant and with sharp edges). This technique has been widely used in the USA while some researchers have even employed remote sensing imaging^46^. We sampled within 5–30 km north and 5–80 km south of Perth city. We categorized public spaces by size (small [0.5–1 km^2^], medium [1.1–2 km^2^], large [2.1–2.9 km^2^], and extra-large [≥3 km^2^]) based on area data from local council authority websites in each study area or, where no information was available, we estimated the area of the space in relation to other known areas. All the public spaces that met the size cut off and had a grass variety that was commercially available were included in the sampling frame, however, sampling was performed on the basis of convenience. We estimated that 150 samples were appropriate to detect 18% difference in *C. difficile* prevalence between NL and OL (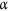 = 0.05, power 80%, 95 CI). To account for sampling clustering, we multiplied 150 samples obtained by simple random sampling by 2 design effect. A total of 311 NL or OL samples were collected in sterile 200 mL specimen jars. Each public space was divided into four quadrants with the centre generally being a children’s play facility. Four samples were obtained per quadrant starting from the centre and moving outwards. The lawn sample consisted of grass and its root system with attached soil, and was obtained using a new set of sterile examination gloves and a sterile tongue depressor which was used to dig out grass and its root system. Each lawn sample weighed approx. 50 g and these were transported at ambient temperature to the laboratory.

### *C. difficile* isolation

Ninety mL brain heart infusion broths supplemented with cycloserine and cefoxitin, were pre-reduced for 4 h in an anaerobic chamber (A35, Don Whitley Scientific Ltd., Shipley, West Yorkshire, UK) at 37 °C. The lawn samples were aseptically cut into ~5 cm^2^ pieces weighing approx. 5 g, inoculated into the broth and gently shaken, and then incubated at 35 °C in an A35 Anaerobic Workstation (Don Whitley Scientific Ltd., UK) for 5 days with the lids loose. A 5 mL aliquot of culture broth was mixed with 5 mL anhydrous ethanol (96%) and incubated for 1 h before being centrifuged at 3000 *g* for 10 min. The pellet was plated onto C. diff ChromID agar (bioMérieux, Marcy l’Etoile, France) and the plates incubated anaerobically for 48 h. *C. difficile* was identified on the basis of its characteristic chartreuse fluorescence detected with UV light (~360 nm wavelength), morphological characteristic (ground glass appearance) and odor (horse dung)^32^. Identification of uncertain isolates was achieved by Gram staining and detection of L-proline aminopeptidase (Remel Inc., Lenexa, KS, USA).

### Quantitation of *C. difficile* in lawn samples

A viable count of *C. difficile* was performed on a subset of lawn samples using previously described methods with some modifications^9^,^34^. Briefly, approx. 5 g of soil was aseptically obtained from an approx. 10 g lawn sample by shaking the root system and placed in a Stomacher bag (Colworth, London, UK) containing 25 mL of phosphate buffered saline at pH 7.4 supplemented with 0.1% Tween 20 (PBST) or peptone saline also containing 0.1% Tween 20 (PST) and shaken for 60 s. A 100 μL aliquot was removed and directly inoculated onto C. diff ChromID agar, spread using a sterile hockey stick (InterPath Services Pty Ltd, West Victoria, Australia) and incubated anaerobically. The remaining volume was centrifuged at 3000 *g* for 10 min, the supernatant discarded, the pellet resuspended in 1000 μL of either PBST or PST and 100 μL directly inoculated on C. diff ChromID. Viable colony counts were performed at 48 h. A negative control (either PBST or PST) was used in each assay to monitor for potential contamination. We performed molecular typing on all positive samples using toxin PCR and PCR-ribotyping to confirm the isolates. The detection limit for the viable count was >1 CFU per gram of soil.

### Molecular characterization

All isolates were characterized by PCR to determine the presence of toxin A (*tcdA*), B (*tcdB*), and binary toxin (*cdtA* and *cdtB*) genes^47^,^48^. Novel primers were multiplexed with tcdA primers NK2 and NK3^46^ to detect the *tcdA* repeat region A3 fragment. Briefly, 4 μL of DNA extract was added to a PCR mixture containing 2 mM MgCl_2_, 50 mM KCl, 10 mM Tris–HCl (pH 8.3), 200 mM of dNTP, 0.2 mM of each primer NK2, NK3 and BE*-tcdA-1* (FP: 5′CAGTCACTGGATGGAGAATT-3′ and BE*-tcdA-2* (RP: 5′-AAGGCAATAGCGGTATCAG-3′ [B. Elliott, unpublished data]. Detection of both the *tcdA1* and *tcdA3* fragment was required for an isolate to be considered *tcdA* positive. Amplifications were carried out in a 2720 Thermal Cycler (Applied Biosystems, Foster City, USA).

PCR ribotyping was performed on isolates as described elsewhere^49^. RTs were identified by comparing their banding patterns with those in our reference library from the Anaerobe Reference Laboratory (ARL, Cardiff, UK) ribotypes that included 15 reference strains from the European Centre for Disease Prevention and Control (ECDC) and the most prevalent PCR ribotypes currently circulating in Australia (B. Elliott, T. V. Riley, unpublished data). Isolates that could not be identified with the international reference library were designated with local (QX) nomenclature.

### Statistical analysis

Univariable random effect logistic regression analysis was used to describe the relationship between *C. difficile* culture status (response variable), and independent variables: age of lawn (new vs. old), sampling site, location (north vs south), postcode, size of playground (small, medium, large, and extra-large) and season (autumn: March, April, May and winter: June, July August). Two random effects, postcode and sampling site were included in the model to account for clustering or autocorrelation among samples from the same sampling site and postcode. Univariable models were constructed including one main effect and two random effects per model, then a backward stepwise multivariable model including the interactions terms, main effects and random effects. In the multivariable model, variables that were not statistically significant at 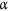 = 0.05 were excluded from the model with the assumptions that they were not confounders. Variables were retained in the model if they were significant or part of their interaction terms was significant or they were considered as confounders. Random effects were excluded from the model if they explained very little from the models based on the change in Akaike’s Information Criterion (AIC), Bayesian Information Criterion and deviance *χ*^2 ^50. Model selection was also evaluated by the likelihood ratios using the generalized linear model (GLM) framework using R version 3.3.1.

## Additional Information

**How to cite this article:** Moono, P. *et al*. High prevalence of toxigenic *Clostridium difficile* in public space lawns in Western Australia. *Sci. Rep.* **7**, 41196; doi: 10.1038/srep41196 (2017).

**Publisher's note:** Springer Nature remains neutral with regard to jurisdictional claims in published maps and institutional affiliations.

## Figures and Tables

**Table 1:** The relationship between the prevalence of *C. difficile* in lawn and the age of the lawn, its size, sampling site, location, postcode, and season in Perth.

Variable	Variable categories	*C. difficile* number isolated (%)	Univariable model	Covariate Odds ratios (95% CI)*	
			Odds ratios (95% CI)†	Sampling site	*P value^¶^*
Age‡	Old lawn (n = 113)	53 (47)	Referent		
	New lawn (n = 198)	129 (65)	2.11 (1.32–3.4)	2.30 (1.16–4.57)	0.015^#^
Area	Extra-large (n = 85)	53 (62)	Referent		
	Large (n = 53)	26 (49)	0.58 (0.28–1.16)	0.49 (0.16–1.49)	0.7
	Medium (n = 101)	60 (59)	0.88 (0.49–1.59)	1.02 (0.42–2.51)	0.7
	Small (n = 72)	43 (60)	0.89 (0.47–1.71)	0.88 (0.32–2.43)	0.7
Location	North (n = 161)	98 (60.9)	Referent		
	South (n = 150)	84 (56)	1.22 (0.78–1.92)	1.25 (0.61–2.59)	0.99
Season	Autumn (n = 224)	135 (60.3)	Referent		
	Winter (n = 87)	47 (54)	0.77 (0.47–1.28)	0.67 (0.28–1.62)	0.52

^*^CI; The 95% confidence interval of odds ratio for the covariate estimates.

^†^CI; The 95% confidence interval of odds ratio for the univariate estimates.

^‡^Age of lawn; new lawn ≤4 months, old lawn >4 months.

^¶^P values are based on likelihood ratios and *P < 0.05* was considered significant^#^. Univariable logistic regression model with random effect (site of sampling and postcode). The random effect term for postcode was not included in all the models because its addition or removal did not change the model estimates significantly.

**Table 2:** Viable counts of *Clostridium difficile* in soil from lawn samples using either phosphate buffer solution (pH 7.4) (PBST) or peptone saline (PST), both containing 0.1% Tween 20, as a diluent.

Diluent	Sample (viable count cfu/g of soil)										No. samples positive
	1	2	3	4	5	6	7	8	9	10	
PBST	0	0	0	50	50	250	0	0	0	100	4
PST	0	0	0	800	200	100	0	50	0	1200	5

**Table 3:** The frequency and toxin gene profile of *C. difficile* ribotypes in lawns in Perth, Western Australia.

Ribotype	Toxin gene profile			Number (%)
	*tcdA*	*tcdB*	*cdtA/B*	
014/020	**+**	**+**	−	71 (39.0)
010	−	−	−	37 (20.3)
QX 077	−	−	−	13 (7.1)
QX 189	−	−	−	7 (3.9)
039	−	−	−	5 (2.7)
QX 601	−	−	−	4 (2.2)
393	−	−	−	4 (2.2)
QX 142	−	−	−	4 (2.2)
002	**+**	**+**	−	4 (2.2)
Others*	Various	Various	−	33 (18.1)

^*^Others: QX 518, QX 611, QX 608, QX 610, RT 056, RT 054, QX 607, QX 606, QX 605, QX 603, QX 602, QX 550, QX 449, QX 409, QX 393, QX 210, RT 125, QX 121, RT 106, QX 072, QX 067, QX 054, RT 018, RT 012, RT 009 and RT 043.
